# Similarities and Differences between Gas Diffusion Layers Used in Proton Exchange Membrane Fuel Cell and Water Electrolysis for Material and Mass Transport

**DOI:** 10.1002/advs.202309440

**Published:** 2024-06-18

**Authors:** Tao Zhang, Ling Meng, Chengcheng Chen, Lei Du, Ning Wang, Lixing Xing, Chunmei Tang, Jian Hu, Siyu Ye

**Affiliations:** ^1^ Huangpu Hydrogen Energy Innovation Center School of Chemistry and Chemical Engineering Guangzhou University Guangzhou 510006 China; ^2^ China Electronic Product Reliability and Environmental Testing Research Institute (CEPREI) Guangzhou 510610 China; ^3^ School of Light Industry and Engineering South China University of Technology Guangzhou 510640 China; ^4^ SinoHykey Technology Company, Ltd. Guangzhou 510 760 China

**Keywords:** gas diffusion layer, mass transports, materials, proton exchange membrane fuel cell, proton exchange membrane water electrolysis cell

## Abstract

Proton‐exchange membrane fuel cells (PEMFCs) and water electrolysis (PEMWE) are rapidly developing hydrogen energy conversion devices. Catalyst layers and membranes have been studied extensively and reviewed. However, few studies have compared gas diffusion layers (GDLs) in PEMWE and PEMFC. This review compares the differences and similarities between the GDLs of PEMWE and PEMFC in terms of their material and mass transport characteristics. First, the GDL materials are selected based on their working conditions. Carbon materials are prone to rapid corrosion because of the high anode potential of PEMWEs. Consequently, metal materials have emerged as the primary choice for GDLs. Second, the mutual counter‐reactions of the two devices result in differences in mass transport limitations. In particular, water flooding and the effects of bubbles are major drawbacks of PEMFCs and PEMWE, respectively; well‐designed structures can solve these problems. Imaging techniques and simulations can provide a better understanding of the effects of materials and structures on mass transfer. Finally, it is anticipated that this review will assist research on GDLs of PEMWE and PEMFC.

## Graphical abstract

This review mainly introduces the differences and similarities of GDL between the PEM fuel cell and electrolyzer, including material and mass transport. Carbon and metal materials are investigated for the two devices, but there are different focuses. There is a great correlation between structures and the mass transports, which eventually affects the two cell performance.

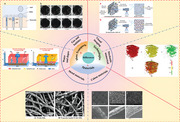

## 1 Introduction

**Figure 1:**
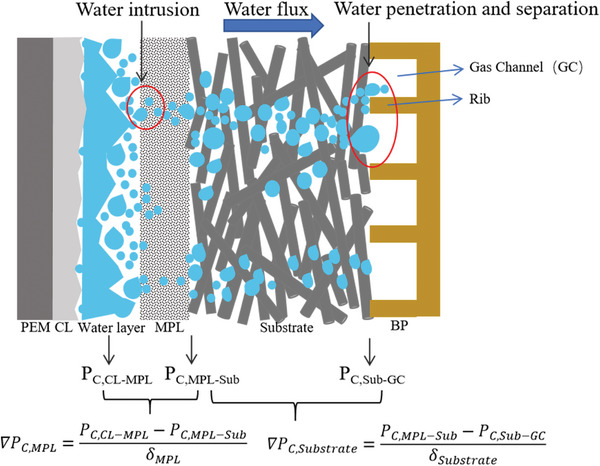
Scheme of water transport process and driving force in single‐side cell. The driving force in the passages is applied by the capillary pressure difference. ∇*P*_C, MPL_ and ∇*P*_C, Substrate_ are capillary pressure differences: the interface of CL/MPL and MPL/MPS, as well as MPL/MPS and MPS/GC (MPL is a microporous layer, MPS is a macroporous substrate and GC is a gas channel).

Recently, clean and renewable energy resources have attracted considerable attention owing to global energy consumption and environmental issues. Under the low‐carbon target, hydrogen energy has been paid more and more attention because of its high efficiency, clean, and sustainable source. Among these technologies, two proton exchange membrane electrochemical devices (i.e., proton exchange membrane fuel cell and electrolyzer) are promising devices for hydrogen production and utilization. Both electrochemical devices have similar membrane electrode assembly (MEA) components: bipolar plates (BPs), gas diffusion layers (GDLs), catalyst layers (CLs), and proton exchange membranes (PMEs). Among these components, the GDLs placed between the CL and BP, are significant and play multiple identical and important roles. First, it must possess high electronic conductivity to form a perfect electrical connection, and the reaction heat should be effectively removed away from the catalyst layer: consequently, GDLs should have good thermal conductivity. Furthermore, it provides strong mechanical support for the membrane electrode assembly (MEA). Most importantly, the GDL provides sufficient transport passages for gases (i.e., hydrogen and oxygen) and water. The approximate water transport process and driving force in a single‐sided PEMFC are displayed in **Figure** **1**. The combined effects depend on the materials and structures the of GDL.

**Figure 2:**
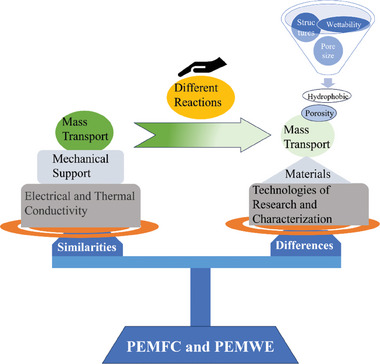
The differences and similarities between PEMWE and PEMFC.

Remarkably, the GDLs of both PEMWE and PEMFC have similarities, but have differences in materials and mass transfer characteristics, as displayed in **Figure** **2**. Therefore, this review will introduce the differences and similarities between the GDL of PEMWE and PEMFC. Both the anode and cathode GDL of PEMFC commonly use carbon materials.^[^ ^1^, ^2^ ^]^ For example, Yang et al. used carbon paper as the anode and cathode substrates for fuel cells to study the root cause of GDL degradation.^[^ ^1^ ^]^ Of course, metal substrates improve GDL corrosion resistance.^[^ ^3^ ^]^ For PEMWE, the cathode potential is lower than that of the anode, consequently, carbon paper can be used, significantly reducing raw material costs.^[^ ^4^, ^5^ ^]^ So it can be known that carbon materials are not only widely used in PEMFC, but also in PEMWE cathode. Instead, metallic materials, especially Ti metal, are the most favored materials for PEMWE.^[^ ^6^, ^7^ ^]^ In addition, metals such as stainless steel and nickel have been explored.^[^ ^8^, ^9^ ^]^ In response to the microporous layer (MPL), PEMWEs are usually coated with precious metals and their oxides to enhance the corrosion resistance.^[^ ^10^ ^]^ Conversely, the MPL in PEMFC is mixed with carbon black and polytetrafluoroethylene (PTFE) to enhance the electrical conductivity and improve water management.^[^ ^6^ ^]^

**Figure 3:**
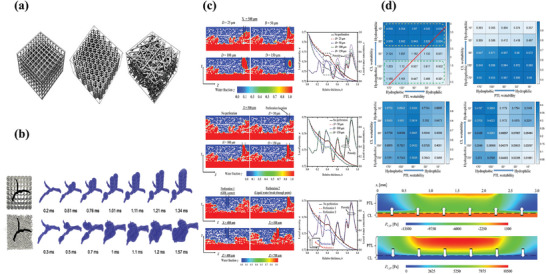
a) Microstructures of GDLs. Reproduced under terms of the CC‐BY license.^[^ ^12^ ^]^ Copyright 2019, Daniel Niblett, published by ECS. b) Dynamic drainage process of two structures: anisotropic lattice structure and reconstructed GDL sample. Reproduced with permission.^[^ ^11^ ^]^ Copyright 2020, Elsevier. c) 2D contours of liquid water fraction in GDL with different perforation diameters, depths, locations; and local porosities and liquid water saturations. Reproduced with permission.^[^ ^19^ ^]^ Copyright 2019, Elsevier. d) Effect of PTL and CL wettability on cell: current density under different wettability conditions @2 V; gas volume fraction in the anode CL under different wettability conditions @2 V; gas volume fraction in the anode PTL under different wettability conditions @2 V; gas volume fraction in the anode channel under different wettability conditions @2 V; gas capillary pressure distribution in anode PTL and CL @ contact angle CL  =  90°, PTL  =  130°; gas capillary pressure distribution in anode PTL and CL@ contact angle CL  =  90°, PTL  =  50°. Reproduced with permission.^[^ ^23^ ^]^ Copyright 2022, Elsevier.

Mass transport issues must be addressed continuously. Various structural modifications of GDL have been attempted to achieve good mass transport management. In PEMFCs, the GDL is composed of nonuniform carbon fibers with different microstructural layers in PEMFC and the pore structure in each layer of the carbon fiber cannot remain the same. However, surprisingly, the ordered structure can guide water flow, which makes it easy to characterize the relationship between the microstructure and gas‐water dynamics. Niblett et al. built an ordered GDL structure of GDL to realize a uniform arrangement of pores using simulation modeling (**Figure** **3a**). They demonstrated that ordered structures improve the effective electrical conductivity, and the presence of defects in the MPL causes saturation of water pathways, acting as water conduits and improving access to oxygen in the GDL and MPL (Figure 3b).^[^ ^11^, ^12^ ^]^ Balakrishnan et al. developed a GDL with gradients from the CL to the gas channel. Their results concluded that the pore gradient structure enhanced the performance of effective and directed water removal, leading to a lower mass transport resistance in PEMFC.^[^ ^13^ ^]^ The large or small cracks formed naturally on the surface of the MPL after drying.^[^ ^14^ ^]^ These cracks are usually the initial water transport passages and partly benefit the expulsion of water. To manufacture human‐made cracks, perforation is a good way to realize an ordered and arrayed pore structure, which is achieved using a thermally decomposable pore agent,^[^ ^15^ ^]^ a laser‐based technique,^[^ ^16^, ^17^ ^]^ and mechanical processes.^[^ ^18^ ^]^ The position and depth of the perforation and pore shape deserve more attention. Niu et al. reported that GDL perforation should be near the water breakthrough point.^[^ ^19^ ^]^ This can facilitate the removal of liquid water to prevent coverage of the entire front region.^[^ ^20^ ^]^ Simultaneously, Niu showed that the deeper perforation depth can reach the liquid waterfront and that perforating the entire GDL was not necessary (Figure 3c). In the PEMWE, the Ti meshes are the cheapest GDLs; however, the performance of the electrolyzers with this type of GDL is not as high as that of sintered structures or felts.^[^ ^21^ ^]^ Kim et al. tailored a bilayer Ti mesh porous transport layer to enhance the performance of stacked bilayers with different pore sizes. The smaller pore closing to the CL caused the highest mass transport losses because the bubbles filled up the pores and the large pore size prevented the bubble's separation.^[^ ^22^ ^]^ Besides, owing to the different materials used in the GDL of PEMWE and PEMFC, the pore size of the PEMWE is always larger than that of the PEMFC. Therefore, gas is more easily transported through large pores. However, the specific behavior of bubbles inside pores has not yet been clearly demonstrated. Water transport in PEMWE has not been extensively studied and reported.

Wettability is another factor that affects mass transport. For different transport goals in the fuel cell, the macroporous substrates (MPS) experience a hydrophobic treatment process, achieving more effective water transport functions. Tamayol et al. immersed the carbon paper with different PTFE contents. They found that the permeation threshold increased with an increase in PTFE loading. This indicates that water was not trapped in the pores.^[^ ^24^ ^]^ Mortazavi et al. reported that PTFE caps the separation diameters of the dope.^[^ ^25^ ^]^ In addition, Lim et al. reported that the PTFE could change not only the wettability but also the porosity of the GDL.^[^ ^26^ ^]^ Therefore, when producing the carbon paper, the porosity and pore size must be limited. The distribution of PTFE is considered a question, and Lim indicated that a high PTFE content in the outlet of the GDL benefits water drainage. In contrast, the hydrophilic GDL shows better performance, as the reactant (water) can provide a sufficient supply to the reaction sites.^[^ ^27^ ^]^ Jiang et al. assembled two MEAs with a hydrophobic CL and hydrophilic GDL, as well as a hydrophobic GDL and hydrophilic CL. The final results demonstrated that the combination of a hydrophilic CL and hydrophobic GDL showed a higher performance than the other combinations (up to 12.6 times). The capillary pressure from the CL to the GDL can promote gas discharge (Figure 3d).^[^ ^23^ ^]^ Unlike PEMFC, hydrophilic GDL indeed performed better than hydrophobic GDL in PEMWE.^[^ ^27^ ^]^ This paper reviews the differences and similarities between the material and mass transport characteristics of the GDLs used in PEMWE and PEMFC. Finally, the challenges of PEMWE and PEMFC include the GDL material cost and preparation cost of PEMFC and PEMWE, as well as the corrosion resistance of the GDL materials in PEMWE. The nanoscale micropores inside the GDL are still clearly characterized; thus, the specific impact of these pores on mass transfer is unclear. Researchers have studied gas‐liquid transfer by constructing a transparent GDL, which is a good reference and can provide a visual understanding of the mass‐transfer state. Although GDL has been commercially applied for some time, some problems mentioned in this review should be overcome as soon as possible to accelerate the transformation of experimental research results to commercial applications of GDL in PEM devices.

## 2 Differences and Similarities of GDL Materials for PEMFC and PEMWE

### 2.1 Carbon‐Based and Metal‐Based Materials in Macroporous Substrates

#### 2.1.1 Carbon‐Based Materials

**Figure 4:**
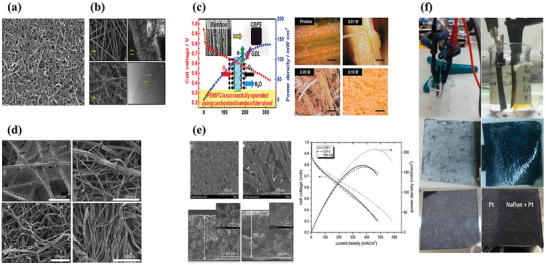
a) SEM of CNT paper. Reproduced with permission.^[^ ^29^ ^]^ Copyright 2015, Elsevier. b) Morphology of carbon fiber paper with MWCNTs and the TEM image of MWCNTs. Reproduced with permission.^[^ ^2^ ^]^ Copyright 2019, Elsevier. c) Results for PEMFC operation tests employed CBFS (squares) and CP (circles) as GDL for the cathode; and optical microscopic images of the pristine bamboo strips and after delignification. Reproduced with permission.^[^ ^30^ ^]^ Copyright 2015, American Chemical Society. d) SEM images of different carbonized GDL materials: carbon fiber, Jute, Cotton, and Muslin. Reproduced with permission.^[^ ^31^ ^]^ Copyright 2022, Elsevier. e) Cross section and surface morphologies of Coconut Carbon Production, and Cell MEA performance. Reproduced under terms of the CC‐BY‐ND license.^[^ ^32^ ^]^ Copyright 2018, Fredina Destyorini, published by ITB Journal Publisher. (f) Images of the electrophoretic deposition system and the GDL covered by TiO_2_, Nafion, and Pt, respectively. Reproduced under terms of the CC‐BY license.^[^ ^33^ ^]^ Copyright 2019, Silvia Scalese, published by Materials and Devices for Solar to Hydrogen Energy Conversion.

Carbon‐based and metal‐based macroporous substrates (MPS) used both PEMWE and PEMFC. Generally, carbon fibers are mostly produced from precursor polyacrylonitrile (PAN) pyrolysis and can be shaped into various types (i.e., typical carbon paper, cloth, and felt). Carbon papers are the most widely used materials in GDL for PEMFC. Similarly, carbon fiber substrates are only used in the cathode of the PEMWE, which is inappropriate for the anode of the PEMWE because the anode potential is much higher than the cathode potential. This is the main reason for the material differences. The carbon materials of the MPS between the PEMFC and PEMWE are listed in **Table** **1**. PAN‐based GDL with mature preparation processes exhibits more advantages over other materials, such as high gas permeability and smooth surfaces. In recent years, it has been difficult to improve the performance of PEMFC by changing only the carbon fiber for the MPS. Therefore, the carbon additives are commonly used in MPS. For example, Kaushal et al. added natural graphite to normal carbon fibers to form novel carbon fibers that exhibited new material properties with increased bulk density and lower in‐plane electrical resistance in fuel cells.^[^ ^28^ ^]^ Similarly, they incorporated carbon nanotubes (CNTs) with carbon fiber paper (**Figure** **4a**) in two ways to modify the structures and materials; more details are provided in reference_._^[^ ^2^ ^]^ Deng et al. produced an MWCNT‐based MPS that demonstrated a maximum power density 45% higher than that of the MPL‐coated commercial TGP‐H‐090 (Figure 4b).^[^ ^29^ ^]^ However, CNT additives used in MPS are difficult to commercialize because the high cost of raw materials is one of the reasons limiting the fabrication of CNTs‐based MPS.

**Table 1:** The carbon and metal‐based materials of MPS in the PEMFC and PEMWE.

Materials		Reference in PEMFC	Reference in PEMWE	
Carbon‐based materials	PAN‐based carbon materials	Carbon paper^[^ ^42^, ^43^, ^44^ ^]^ Carbon cloth^[^ ^44^, ^45^ ^]^ Carbon felt^[^ ^44^, ^46^ ^]^	Cathode	Carbon paper^[^ ^10^, ^33^, ^40^, ^47^ ^]^ Carbon cloth^[^ ^48^ ^]^
	Pitch‐based carbon materials	^[^ ^49^, ^50^ ^]^	–	
	Natural plant carbon materials	Jute, Cotton, Sisal, Sugarcane bagasse, and Switchgrass fiber,^[^ ^31^ ^]^ Bamboo fiber,^[^ ^30^ ^]^ Coconut coir fiber^[^ ^32^ ^]^	–	
Metal‐based materials	Ti	Ti foam^[^ ^51^ ^]^ Ti thin film^[^ ^3^ ^]^	Anode	Ti powder^[^ ^52^ ^]^ Titanium sinter Plates^[^ ^53^ ^]^ Ti Mesh^[^ ^54^ ^]^ Ti felt^[^ ^4^, ^7^ ^]^
	Stainless steel	^[^ ^55^ ^]^		^[^ ^3^, ^9^ ^]^
	Other metals	Ni mesh^[^ ^56^ ^]^ Cu foil^[^ ^57^ ^]^		Ti/Cu/Al powder^[^ ^52^ ^]^ Ni foam^[^ ^58^ ^]^

In addition, it must be noted that PAN fibers bring about relatively high production costs for the GDL, which contribute to the high cost of the MEA. Therefore, sustainable natural plant fiber materials, such as cellulose,^[^ ^34^ ^]^ coconut fibers,^[^ ^35^ ^]^ and other biomass‐derived carbon fibers^[^ ^30^ ^]^ are also being explored as precursors to manufacture GDL substrates reducing the costs of the initial materials (Figure 4c). For example, Leonard et al. used rows, unbleached jute, cotton, sisal, sugarcane bagasse, and switchgrass fibers as the precursors to form a substrate layer (Figure 4d).^[^ ^31^ ^]^ Through the densification step and gas‐phase hydrophobic treatment with the trichlorosilane. This GDL formed two promising GDL architectures, one using a fabric and paper layer, and the other utilizing two fabric layers whose performance was similar, but their performance is difficult to match with that of commercial carbon paper (TGP‐H‐120). Similarly, other carbon‐particle materials and biomass carbon fibers can be mixed to prepare carbon paper. For example, Destyorini et al. fabricated a substrate layer using carbon powder and carbon fibers from coconut coir (Figure 4e). Comparing the comparable porosity, average pore size, and water contact angle of the newly fabricated MPS and Toray TGP‐H‐120, the electrical conductivity of the new MPS was much lower than that of the commercial PAN carbon paper. So, its maximum PEMFC power density was 40 mW cm^−2^ lower than that using TGP‐H‐120 a PAN‐based carbon paper.^[^ ^32^ ^]^ Pitch‐based carbon fibers are another alternative to PAN‐based carbon fibers, mainly because of their high modulus, high thermal conductivity, and carbonization rate of over 75%. However, plant‐derived and pitch‐based carbon fibers have low electrical conductivities.^[^ ^36^ ^]^ Thus, Heo et al. added Ketjenblack (with the advantages of high surface area and good conductivity) to a pitch‐based MPS to enhance electrical conductivity. To reduce production costs from the high temperature (1000–1400 °C), Heo and his colleagues specifically investigated the performance of pitch‐based carbon fiber papers after the heated treatment with a temperature below 800 °C. The results indicated that with an increase in the carbonization temperature and Ketjen black contents, the electrical conductivity was in a range of (2.587–2.641) **×** 10 S cm^−1^.^[^ ^37^ ^]^ However, they did not operate it in a PEMFC to show its electrochemical performance; perhaps, its electrical conductivity is much lower than that of commercial carbon paper (660 S cm^−1^) one of the reason. Therefore, pitch‐based carbon materials require further investigation.

For PEMWE, it experiences the reaction that water is decomposed to hydrogen and oxygen through the electricity, consequently, the materials must keep high electrochemical corrosion resistance to achieve long stability. Therefore, the anode materials are always metals. In single‐cell tests, the cathode GDL can utilize the conventional carbon fiber MPS commonly used in PEMFC, which can provide better performance than the metal‐based GDL in the cathode under a low potential because carbon fibers can easily form easier to form the proper porous structures.^[^ ^38^ ^]^ Certainly, some research groups^[^ ^39^, ^40^, ^41^ ^]^ have investigated carbon MPS assembled on the anode side, yet. Filice et al. covered a thin Pt protective layer on anode and cathode PAN carbon paper substrates (Figure 4f), which effectively improved the durability.^[^ ^33^ ^]^ The addition of a coating is the most conventional approach for protecting the substrate layer in PEMWE. For example, in 2009, Ma et al. used carbon paper as the MPS and added a protective MPL mixed with IrO_2_ and Ti powder. Although these coatings can provide some protection to slightly extend their lifetime, they can only work in the short term, and their operating conditions must be closely monitored.^[^ ^40^ ^]^

This section introduces various GDL materials, including PAN carbon fibers, natural plant carbon fibers, and pitch‐based carbon fibers, which are typically made of carbon paper, cloth, and felt. All these materials have been investigated in PEMFC, and PAN carbon papers are the most widely used GDL because they provide better water and gas management than the GDL of other materials. GDLs made of other carbon materials are positive attempts to provide more guidance for future research on the gas diffusion layer. However, these materials have some defects, such as low electrical conductivity, low strength, and high brittleness, which affect the MEA assembly and its performance. According to the literature almost all PEMWE cathodes use commercial GDL (i.e., PAN carbon paper, cloth, and felt commonly used in PEMFC) to transport gas and water. At the anode, the addition of a metal protective layer allows the carbon material GDLs to realize short‐term operation. Thus, the PEMFC and the cathode of the PEMWE can use the same carbon GDLs, but the anode of the PEMWE must utilize metal materials, as carbon materials cannot withstand high potentials.

#### 2.1.2 Metal‐Based Materials

**Figure 5:**
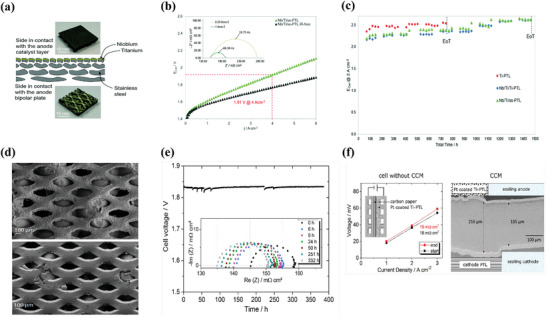
a–d) Image of the four‐layers ss‐mesh PTL with a plasma‐sprayed Nb/Ti coating; Polarization curve and the iR‐free curve up to 6 A cm^−2^ of a PEMWE cell with Nb/Ti/ss‐PTL; accelerated stress test (AST) of cells with the Ti‐PTL, Nb/Ti/Ti‐PTL and Nb/Ti/ss‐PTL; SEM contrast images of ss‐PTL before and after the AST. Reproduced under terms of the CC‐BY license.^[^ ^9^ ^]^ Copyright 2022, Svenja Stiber, published by Royal Society of Chemistry. e,f) Cell voltage and impedance spectra during galvanostatic operation at 2 A cm^−2^; Ohmic resistance of the cell when assembled without the CCM before and after the test; and Cross‐section of the CCM after the test. Reproduced with permission.^[^ ^53^ ^]^ Copyright 2016, Elsevier.

As mentioned above, the metallic materials are more prevalent in anode PEMWE than in carbon‐based GDLs. The metallic materials of the MPS between the PEMFC and PEMWE are listed in Table 1. First, stainless steel, which is a low‐cost material, naturally serves as a substitute material, which some research teams have investigated.^[^ ^9^ ^]^ Lettenmeier et al. fabricated a porous Ti layer on the surface of stainless steel.^[^ ^59^ ^]^ Regrettably, this paper introduces the role of sintered Ti plates fabricated using vacuum plasma spraying (VPS) technology. Stiber et al. compared Ti and stainless steel with and without coatings (**Figure** **5a**). The results showed that GDLs with coatings showed high performance in the PEMWE (1.9 V @ 4 A cm^−2^ at 1 A cm^−2^) (Figure 5b). After >1000 h at AST, the Nb/Ti metal coating protected the stainless‐steel GDL against corrosion (Figure 5c,d).^[^ ^9^ ^]^ The results of this study demonstrate that stainless steel is promising for PEMWE because its performance is comparable to the advanced performance of Ti‐based GDL. Therefore, low‐cost stainless steel can be used to fabricate the GDL. Stainless steel has also been used to investigate the passivation and corrosion mechanisms of metal migration.^[^ ^3^, ^60^ ^]^ Mo et al. intentionally placed stainless steel at the anode to study the corrosion mechanism of metal migration. They documented the degradation of 316 stainless‐steel mesh in the single‐cell PEMWE operated at a current density of 1 A cm^−2^ with a cell voltage of 2.8 V for 15 h. Then the corroded products (mainly Fe_3_O_4_ and NiO) were found in the cathode by SEM and STEM‐EDX.^[^ ^3^ ^]^ This phenomenon also proves that even though it is stainless steel without a protective coating, the GDL will still be corroded.

A GDL is required to provide a low interface contact resistance, strong mechanical strength and support, and good thermal and electrical conductivity. In addition, the GDL inhibits the degradation or formation of passivating layers under a corrosive environment for a long time. Unlike carbon paper and stainless steel, Ti (as a representative of “valve” metals) exhibits a high corrosion resistance, superior mechanical properties, and low density and is thus currently the most suitable substrate material for PEMWE. There are several shapes of Ti for GDL: Ti mesh,^[^ ^5^, ^61^ ^]^ Ti felt,^[^ ^62^ ^]^ and sintered Ti powder.^[^ ^63^, ^64^ ^]^ Based on various considerations, such as the operation environment, performance, and mass transport of PEMWE, the proper selection of material styles for the GDL is significant. Ti fibrous materials have gradually replaced the sintered GDL materials. A research group reported that Ti felt has better oxygen transport properties and a lower cell overvoltage.^[^ ^65^ ^]^ Kang et al. also obtained similar results in that a sintered Ti GDL had a higher cell voltage (or high mass transport resistance) owing to its larger thickness and lower porosity.^[^ ^66^ ^]^ A review showed that the color of the unprotected Ti GDL turned yellow owing to the formation of a thick oxide layer, which was estimated to be ≈20 nm.^[^ ^67^ ^]^ This phenomenon indicates that Ti is easily oxidized in an oxygen environment. Thus, coating materials are used to protect the substrate, which is usually a precious metal (Au, Pt, or Ir) or a metal oxide (TiO_2_ or IrO_2_). For example, through a 1000 h durability test of two cells, Rakousky and Reimer discussed whether corrosion of the anode Ti GDL (with and without Pt coating on the Ti GDL) was the main source of resistance. The results showed that the cell resistance of the GDL without the Pt coating increased from 49 nm at the beginning to 238 nm at the end for a thick oxide layer. By contrast, the Pt‐coated Ti GDL presented a degradation rate of only 12 µV h^−1^ during a period of 380 h and decreasing ohmic resistance during the first 332 h (**Figure** 5e,f).^[^ ^53^ ^]^ Additionally, Nb may be used in the coatings instead of Pt as an effective solution for protecting Ti substrates.^[^ ^68^ ^]^

Although metal‐based materials such as materials are not competitive in PEMFC compared to carbon‐based materials, they have also been explored in PEMFC because of their excellent mechanical strength and stability at high potentials, such as Ti mesh and foam,^[^ ^69^, ^70^ ^]^ stainless steel^[^ ^55^ ^]^ and nickel.^[^ ^56^ ^]^ Choi et al. successfully fabricated a Ti foam via a freeze‐casting process. This is a promising alternative electrode material for PEMFC because its current density of 462 mA cm^−2^ is ≈166% higher than that of the Toray 060 GDL (278 mA cm^−2^). This Ti foam forming through rapid cooling with liquid nitrogen has a firmly connected 3D strut constriction as well as relatively smaller pores in the range of 10–100 µm, providing improved conductivity, great strength, and sufficient corrosion resistance to maintain the functionality in the acidic solution of the MEA.^[^ ^51^ ^]^ Commercial carbon paper and Ti fiber felts were evaluated as the cathode GDL in PEMFC.^[^ ^71^ ^]^ The results showed that the Ti fiber felts exhibited improved performance owing to their greater porosity and pore size, which contributed to their greater air permeability compared to commercial carbon paper. Recently, there was a research group fabricated a graphite‐coated Ni foam and an ultrathin (9.1 mm) carbon nanofiber film as promising substitutes for conventional channel‐rib flow fields and gas diffusion layers (GDLs). This design significantly reduced the MEA assembly volume (90%) and mass transfer impedance (88.6%) to improve the power density by 50% and achieve advanced PEMFC performance with traditional GDL and flow fields, in which the high porosity of foam Ni was the key to low‐concentration polarization.^[^ ^72^ ^]^

Metal materials provide strong mechanical support, high electrical conductivity, and high corrosion resistance in the complex high‐potential environment of PEMWE and PEMFC, among which Ti is the most prominent, making it the most commonly used metal material and the most researched material, especially in commercial PEMWE. In a relatively lower‐voltage PEMFC, based on the performance exhibited by the metal‐based GDL, the position of the carbon‐based GDL cannot be considered. The reason for the widespread application of metal‐based GDL in PEMFC is limited because their processing and preparation are difficult, and they cannot reach the pore size and porosity of carbon paper. Therefore, further research is required to address these issues and promote the application of metal‐based GDL in PEMFC. Compared with PEMFC, the environment of PEMWE imposes stricter requirements on metal‐based GDL, which require an additional protective layer to be applied. This is an issue that researchers are currently concerned about and the next section will introduce existing protective measures for PEMWE.

### 2.2 Carbon‐Based and Metal‐Based Materials in Microporous Layers

The MPL became a necessary component for the GDL after MPL grades were first manufactured from suspensions of acetylene black and F‐containing polymers by Watanabe^[^ ^73^ ^]^ and later grown by reference.^[^ ^74^ ^]^ In PEMWE, the MPL can be considered as a protective coating. Through this coating (usually precious metals or metal oxides),^[^ ^7^, ^75^ ^]^ the corrosion of the GDL can be reduced and the performance of the PEMWE can be improved, as mentioned earlier. For example, Rakousky et al. found that at a current density of 2 A cm^−2^, the cell performance was stable during constant and intermittent operation. And until up to 3 A cm^−2^ the Pt coating detached from the Ti GDL and adhered to the anode catalyst layer.^[^ ^76^ ^]^ Iridium (Ir) is more commonly used as a GDL coating material because of its good performance and stability compared to Au and Pt in PEMWE. For instance, Liu et al. specifically studied the effects of iridium coatings on a Ti GDL on the performance of a PEMWE. They reduced the loadings of Ir and found that a loading of 0.025 mg cm^−2^ achieved the same cell performance as a GDL with higher Ir loading.^[^ ^77^, ^78^ ^]^ The IrO_2_ and TiO_2_ were grown simultaneously on the surface of the Ti GDL to improve the electron and mass transport and enhance the antioxidant and corrosion resistance in the PEMWE. This method can affect the loading of Ir catalysts, and the results showed that the sample with a load of 0.6 mg_Ir_ cm^−2^ exhibited the highest cell performance at loads of 0.21, 0.39, 0.6, and 0.94 mg_Ir_ cm^−2^.^[^ ^75^ ^]^

In PEMFC, the material composition and role of the MPL are slightly different from those of PEMWE prepared with carbon materials, hydrophobic agents, and organic solvents, and are more focused on promoting mass transfer in PEMFC. Although many hydrophobic agents are available, PTFE remains the most commonly used hydrophobic agent. Organic solvents used as dispersants are usually ethanol and isopropanol, which disperse carbon black particles resulting in an even pore distribution. Finally, the dispersants were removed by heat treatment. Carbon black is the main component of the MPL for PEMFC, providing good physical properties, such as prominent electrical conductivity and excellent contact between the CL and MPS.^[^ ^90^ ^]^ It is generated from the incomplete combustion of coal tar and ethylene‐cracked tar; thus, the costs of raw materials are extremely high and suitable for large‐scale commercial production (**Table** **2**).

**Table 2:** The carbon and metal‐based materials of MPS in the PEMFC and PEMWE.

Materials	Reference in PEMFC	Reference in PEMWE	
Carbon‐based materials	Carbon black (Vulcan XC‐72, acetylene black and Ketjen blacks)^[^ ^79^, ^80^, ^81^, ^82^ ^]^ Carbon nanotubes^[^ ^81^, ^83^ ^]^ Graphite^[^ ^84^, ^85^, ^86^ ^]^	Cathode	Commercial MPL
		Anode	–
Metal‐based materials	–	Nb/Ti^[^ ^9^ ^]^ TiO_2_/IrO_2_^[^ ^75^, ^87^ ^]^ Porous Ti/ Ni^[^ ^8^ ^]^ Ir/Ti^[^ ^40^ ^]^ Pt^[^ ^78^, ^88^ ^]^ Ir^[^ ^41^, ^77^, ^78^, ^88^ ^]^ Au^[^ ^78^, ^88^, ^89^ ^]^	

**Figure 6:**
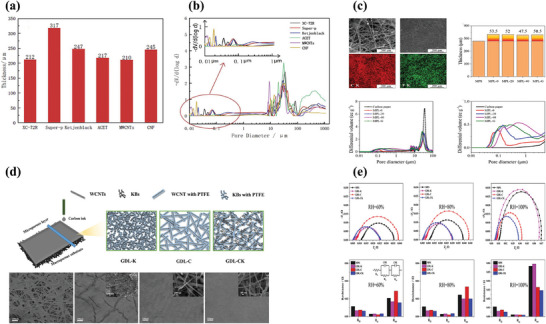
a,b) GDL thickness and pore structure of different carbon materials. Reproduced with permission.^[^ ^91^ ^]^ Copyright 2021, Elsevier. c) Surface SEM images of treated carbon paper and mapping of C‐K and F‐K; thicknesses as well as pore size distribution for different samples. Adapted with permission from.^[^ ^92^ ^]^ Copyright 2020, American Chemical Society. d,e) Schematic diagram of different structural GDLs and surface SEM mapping images; EIS of different GDLs; and dedication of each equivalent circuit component of different MEAs at RH = 60%, 80%, and 100%, respectively. Reproduced with permission.^[^ ^82^ ^]^ Copyright 2022, Elsevier.

Almost all MPL materials are carbon and carbon‐related materials, such as carbon black, graphite,^[^ ^93^ ^]^ graphene,^[^ ^94^ ^]^ and carbon nanotubes,^[^ ^95^ ^]^ all of which can satisfy the requirements of high electrical and thermal conductivity and uniform pore characteristics, and are compatible with commonly commercially employed MPL materials. A series of carbon‐like materials, including Vulcan XC‐72,^[^ ^96^, ^97^ ^]^ Super‐P, Ketjen black,^[^ ^82^ ^]^ and acetylene black (ACET),^[^ ^92^ ^]^ were investigated with MWCNTs to evaluate the distinction between their structures and performances.^[^ ^91^ ^]^ They concluded that the thickness of GDL prepared by Super‐P was the largest and the most reasonable pore structure was in the GDL prepared by ACET that had the most number of pore diameters in the range of 10–30 µm, macropores more than 100 µm, and relatively small nano‐sized pores, as displayed in **Figure** **6a,b**. Chen et al. mixed the ACET and PTFE in a 7:3 ratio by mass to form the MPL, besides, they added pore former with different content to evaluate the effect of the interface at the side of the MPL and catalyst layer (Figure 6c).^[^ ^92^ ^]^ More research has focused on carbon nanofibers and carbon nanotubes, particularly multiwalled carbon nanotubes, with excellent mechanical strength, electronic conductivity, and superior corrosion resistance. In this study, smaller MWCNTs were used to produce MPL instead of MPS. For instance, the reference^[^ ^95^ ^]^ developed two methods for modifying carbon paper with MWCNTs. One was a mixture of MWCNTs and the binder matrix, and then the phenolic resin was introduced into the MWCNT solvent for ultrasonication. In the second case, MWCNTs were grown over a carbon fiber preform by thermal chemical vapor deposition forming a stable and coherent structure by the tangling of nanotubes on the carbon fiber surface. Finally, fibers or particles of different sizes can form different ranges of pore size and porosity, which greatly affect mass transport, as illustrated in a later section. Song et al. applied a combination of MWCNTs and Ketjen black to optimize the pore structure of the MPL.^[^ ^82^ ^]^ A specific network was formed where Ketjen black was evenly distributed around the core MWCNTs, which created more mesopores and resulted in a reasonable pore size distribution; thus, the performance was greatly improved, as illustrated in Figure 6d,e. In addition, Schweiss et al. studied the influence of MWCNTs, in which the porosity remained relatively stable value and prevented the coalescence of carbon black particles. Finally, the modified MPL exhibited a lower tortuosity, which increased the gas permeability.^[^ ^98^ ^]^ The influence of the specific structure influence on mass transport was introduced.

The materials of the MPL in PEMFC and PEMWE are substantially different, with carbon and metal as the main materials for the MPL. The material was optimized in different directions based on the characteristics of the respective operating environments. In PEMFC, water flooding is a key issue affecting GDL performance, and is usually designed by researchers as a pore gradient structure. The preparation of MPL for different carbon materials explored the impacts of carbon black, which is the most mature commercial MPL material and is easier to shape into the desired structure than metal. Surprisingly there are no reports on the use of metal MPL in PEMFC. Evidently, the carbon MPL cannot migrate to the PEMWE for use. In contrast, the metals and metal oxides are widely used as protective layers in PEMWE. In general, carbon and metals are the main materials used for MPL and MPS in both PEMFC and PEMWE. In addition to using a single carbon or metal material, researchers have explored the simultaneous use of two materials (Ni foam and carbon fiber) to prepare GDL.^[^ ^72^ ^]^ It is difficult to significantly improve cell performance by simply changing the preparation materials because the main function of the GDL is mass transfer. In other words, it has been explored from a structural perspective to a greater extent.

## 3 Differences and Similarities of GDL Mass Transfer for PEMFC and PEMWE

With the rapid advancement of catalysts for PEM fuels and electrolyzers, electrochemical reaction kinetics are not always the main constraint on the low performance of stacks; instead, reducing the mass transport limitations becomes more crucial. Because of the opposing reactions of the two PEM devices, the reactants are different, among which water is the reactant of the PEMWE, and hydrogen and oxygen are the reactants of the PEMFC. Thus, the research focuses are different in that the growth and discharge of bubbles are of greater concern in the PEMWE that will produce the “bubbles effect”. Moreover, drainage is required in the fuel cell; otherwise, “water flooding” prevents gas transport. Thus, the structural design and wettability of the GDL of the two devices were distinguished. However, it is worth noting that the gas‐liquid two‐phase mass transfer in the pore is coupled with the PEMFC or PEMWE; therefore, their mass transfer also has commonalities (i.e., gas‐liquid transfer needs to be considered simultaneously). Eventually, the patterns of the GDL were optimized to fit the higher performance requirements.

### 3.1 Gas Transport in PEMFC and PEMWE

One of the most important functions of GDL is the transport and distribution of gases. The gases were removed from the PEM electrolyzers and transported into the PEM fuel cells. However, from existing research, it appears that PEMWE focuses more on gas transportation.^[^ ^99^ ^]^ Before reviewing these research reports, this paper introduces a theoretical formula for gas diffusion. There are two main gas flow regimes: convection and diffusion.^[^ ^100^ ^]^ Gas diffusion depends on the speed of movement and the environment of gas molecules, often occurring in a porous gas diffusion medium. The value of the Knudsen number determines the governing transport types, which are calculated as

(1) $$K_{n}=\frac{\lambda }{d}$$

where *K*_n_ refers to the Knudsen number, λ is the mean free path of flow, and d is the flow channel diameter. When the collision among the gas molecules is more frequent than with the containing walls, *K*_n_ varies in the range of 0.01 to 0.5 which means the flow and diffusion cannot be distinguished. When *K*_n_ > 0.5, the flow dominates over diffusion, which is known as Knudsen diffusion. *K*_n_ < 0.01 indicated that flow transport occurs via permeation, that is, molecular or bulk diffusion. In addition, there is a surface occurs, and gas molecules are absorbed and move along the solid surface. The diffusion of the reactant gases is generally represented by Fick's law^[^ ^101^ ^]^:

(2) $$\varepsilon \frac{\partial c_{i}}{\partial t}=D_{\mathrm{eff}}^{i}\nabla^{2}C_{i}$$

where ε is the porosity of the porous media, *C*_i_ is the concentration of the gaseous species, and *D* ^i^_eff_ is the effective diffusion coefficient of gaseous species. The effective diffusion coefficient is considered an index of the flux of gaseous species through porous media, as it is corrected for the structural restrictions from the porous property of the GDL. The Bruggeman model the is most commonly used gas diffusion model for porous media.^[^ ^102^ ^]^ The Bruggeman relationship usually needs to be modified such that the different magnitudes of n are possibly related to the operating operation conditions and architectures of the GDL, including the water morphology in the pore, as follows:

(3) $$D^{\mathrm{eff}}=D^{\mathrm{bulk}}\varepsilon {\left(1-s\right)}^{n}$$

However, some correlations have overlooked the presence of liquid water in the pores. Subsequently, Das et al. fixed this defect.^[^ ^103^ ^]^ Owejan et al. first reported the effects of water saturation on the effective diffusion coefficient via a direct in situ survey; they obtained accurate exponent “*n*” values in the modified Bruggeman relationship for two GDLs with different diffusion materials (*n* = 3.5 and 4.1).^[^ ^104^ ^]^ Similarly, Chen et al. reported that the effective gas diffusion coefficient exhibited a nonuniform distribution under the channel and rib.^[^ ^105^ ^]^ Besides, Lim et al. took into account the thickness of GDL that defined *β*_avg_, which was integral within the whole thickness.^[^ ^26^ ^]^ The measurement of through‐plane effective diffusivity^[^ ^106^ ^]^ applies the Bruggeman relation, *f*(ε) = ε^1.5^. Tortuosity, τ, is an indicator of the complexity of the diffusion paths that connect the internal pores and compose an intricate network structure. Tortuosity can be transformed into porosity, namely τ = ε^−1/2^. Therefore, Equation (3) can be rewritten as follows:^[^ ^107^ ^]^

(4) $$D^{\mathrm{eff}}=D^{\mathrm{bulk}}\varepsilon^{1.5}{\left(1-s\right)}^{2}$$

**Figure 7:**
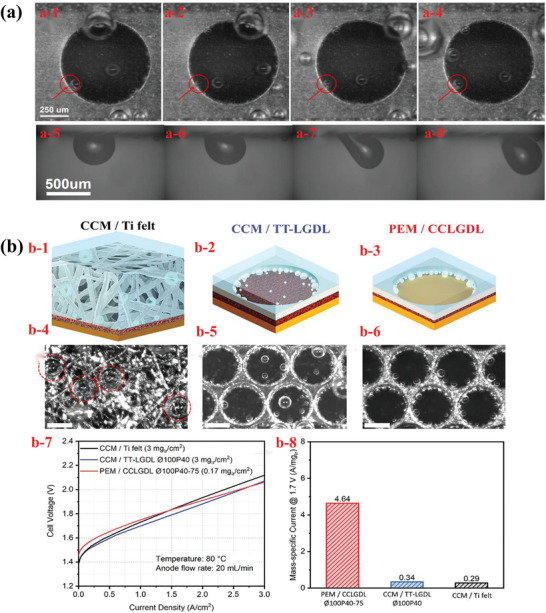
a) The process of bubble detachment on the anode in an operating PEMWE. Reproduced with permission.^[^ ^114^ ^]^ Copyright 2023, Elsevier. b) Images of three different anode electrode designs and corresponding high‐speed video snapshots, polarization curves, and Ir mass‐specific currents (at 1.7 V) of three designs. Reproduced with permission.^[^ ^115^ ^]^ Copyright 2022, Wiley.

Hence, this equation can depict the entire flow in the GDL and can provide accurate results when constructing two‐phase flow models for theoretical calculations. It is essential to gain a 3D realistic structure of the GDL that can determine if the tortuosity is accurate and X‐ray computed microtomography is currently the best imaging technique. However, for porosity, it is easy to decrease when it is in compression^[^ ^105^ ^]^ and to apply a high concentration of PTFE,^[^ ^26^ ^]^ hence, by corresponding experiments the influence of porosity on the effective diffusivity of the GDL can be evaluated. From research reports on the GDL of PEMFC and PEMWE, it was found that the gas effective diffusivity increased as the porosity increased.^[^ ^59^, ^108^ ^]^ For example, Schuler et al. found that for a Ti fiber GDL, as the porosity increased from 56% to 76%, the air permeability doubled.^[^ ^109^ ^]^ Research reports suggest that porosity gradients are more favorable for gas transport in PEMWE.^[^ ^54^, ^72^ ^]^ But the porosity gradient of the GDL in a PEMFC is designed to solve the mass–transfer problem of liquid water. Currently, there are no specialized studies on its impact on gas diffusion. In addition, the pore size is also a significant factor affecting gas transport and presents different results for both PEMWE and PEMFC. In the PEMWE, the mean pore sizes for sintered Ti GDL are in the range from 3 to 6 µm and can achieve a gas permeability of 8 × 10^−13^ to 7 × 10^−12^ m^2^.^[^ ^64^ ^]^ If the pore size is ˂3 µm will make it difficult to expel gas. At this point, the bubble size can be reduced by increasing the operating pressure, thereby discharging it through a small aperture.^[^ ^6^ ^]^ However, in the PEMFC, a pore size of 7–30 µm is advantageous for gas transport possibility owing to its wettability, which is different from that of PEMWE.^[^ ^97^ ^]^ Lin et al. also reported that a pore volume with a pore size of 28.9 µm provides more gas transport space.^[^ ^110^ ^]^ Moreover, Guan et al. demonstrated that in the GDL of PEMFC, water saturation of the channel region from 0% to 15% resulted in a ≈25% reduction in the effective diffusion of oxygen in the channel region, and a weak correlation is shown with the effective diffusion of oxygen of the GDL.^[^ ^111^ ^]^ The large pore size of the GDL on the channel side produced large bubbles that could form a slug flow and hinder water transport. This phenomenon is likely to occur in both the PEMWE and PEMFC.^[^ ^62^, ^112^ ^]^ Different wettability produces different gas mass transfer effects. In the PEMFC, in general, the GDL exhibits hydrophobicity for rapid drainage. Raymond reported that the water saturation distribution in a GDL will affect gas transport. However, most reports indicate that the hydrophobicity of the GDL in PEMWE is not conducive to gas transport. The primary effect of wettability on bubble growth. Hydrophobicity increases, nucleation sites decrease, bubble size increases, and bubbles become difficult to separate from hydrophobic electrodes.^[^ ^113^ ^]^ When bubbles separate from a hydrophobic GDL, a greater pressure drop is required to overcome the resistance caused by hydrophobicity.^[^ ^114^ ^]^ Realistic bubble behavior in the passages of electrolyzers is always the key to research. Selamat et al. revealed two typical cases of gas bubble behavior, quasiperiodic and stagnant, using neutron radiography and optical imaging. The bubbles grew relatively slowly, finally completely occupying the opening, and then passed to the next opening with the water flow. The re‐emergence period was on the order of 1–2 min.^[^ ^95^ ^]^ Wang et al. conducted an in situ visualization experiment in a transparent microchannel to investigate the phenomena of the bubble detachment process. Excess gas induces the attachment of a large number of gas bubbles to attach to the CL and GDL. They then presented four steps of gas bubble detachment: initial phase, instability, deformation, and detachment, as shown in **Figure** **7a**.^[^ ^114^ ^]^ Transparent designs are popular and can be used to study the internal mass‐transfer states of complex microporous structures. Recently, Yu et al. further captured gas accumulation and release beneath the CCLGDL at the reaction interface in a two‐sided transparent reaction‐visible cell. The structural design and high‐speed video snap‐shots are shown in Figure 7b.^[^ ^115^ ^]^

The GDL gas transport in both PEMFC and PEMWE was investigated from two perspectives: experiments and simulations. Experiments are usually accompanied by imaging techniques to characterize the internal structure of the GDL. Unfortunately, the spatial and temporal resolutions of state‐of‐the‐art nano X‐ray imaging technology cannot successfully capture the information on micropores and the gas‐liquid transport status inside the GDL. Therefore, the influence of the pore structure and wettability discussed earlier was mostly characterized by the electrochemical mass–transfer impedance and polarization curves. Additionally, gas transfer research using computational simulation methods is currently has received considerable attention. Pore network modeling (PNM) is commonly used in PEMWE. This can be described by the simulated drainage curve and the capillary entry pressure of the pores.^[^ ^9^ ^]^ And then through the water saturation and the above gas mass–transfer formula, the gas transport characteristics can be calculated. Therefore, both experiments and simulations have been conducted to actively explore the behavior of small bubbles inside the GDLs in both PEMWE and PEMFC.

### 3.2 Water Transport in PEMFC and PEMWE

Water transport has received significant attention but has different requirements for the two devices (i.e., water is transported inward in the PEMWE and discharged outward in the PEMFC). Sufficient supply and uniform distribution of water are the premises of high performance for PEMWE. The above section has mentioned that products (hydrogen and oxygen) significantly block the water supply greatly. Therefore, researchers have adopted various methods for designing the proper structures to promote gas transport. In PEMFC, water flooding always affects performance because of the high mass transport impedance. At a high current density, liquid water is generated heavily, which is difficult to drain, and then blocks the pores available for oxygen and hydrogen transport. This phenomenon is commonly referred to as a “flood”. Optimization of the structural design conducted on the intrusion of MPL and PTFE to form various porosity gradients and wettability areas is the main solution for improving transport in fuel cells. Therefore, the structures and wettabilities of the fuel cells differ from those of electrolyzers.

**Figure 8:**
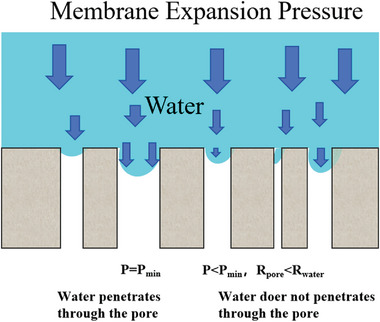
The force applied by membrane swelling. When the swelling pressure is lower than the minimum permeation pressure, the water is hard to penetrate through the pores of MPL. Adapted with permission.^[^ ^116^ ^]^ Copyright 2005, Elsevier.

To remove liquid water successfully and quickly from the MPL/CL interface and internal GDL, the drainage mechanism and specific dynamic behavior of water must first be determined. The removal of water from the MPL/CL interface into the channel typically involves three steps: intrusion, internal transport, and detachment. First, the intrusion of water must overcome the huge resistance of the GDL because treatment with hydrophobic agents results in high surface energy. Therefore, an initial driving force must be present to break through the inlet surface tension. Nehlsen et al. reported that the pressure from the membrane swelling provided the initial driving force (**Figure** **8**). They also indicated that the pressure gradient to drive water flow was much greater than the total surface energy of the hydrophobic pores and the pressure required to drive the flow.^[^ ^116^ ^]^ On the interface, the electrochemical reaction can probably affect the water distribution; however Sarker^[^ ^117^ ^]^ and Molaeimanesh^[^ ^118^ ^]^ have mentioned that a large part of the models is not coupled with the electrochemical reaction and only the microstructure of the GDL. Apart from the surface tension, another key factor is the pore size^[^ ^26^ ^]^ according to the Young–Laplace equation:

(5) $$\Delta P=\frac{2\gamma \cos\theta }{R}$$

**Figure 9:**
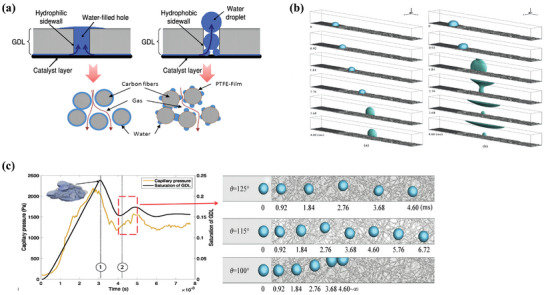
a) Two forms of water flow at different wettability. Reproduced under terms of the CC‐BY license.^[^ ^129^ ^]^ Copyright 2015, Thomas J. Schmidt, published by ECS. b) Droplet dynamic behaviors under novel design strategy of channel and wall. Reproduced with permission.^[^ ^130^ ^]^ Copyright 2020, Elsevier. c) Time‐averaged capillary pressure (yellow line) and GDL saturation profile (black line) during the growth and detachment events; and the increasing of saturation and capillary of GDL at mark 2 due to the droplets detention. Adapted with permission.^[^ ^130^, ^131^ ^]^ Copyright 2020, Elsevier.

The relationship between pore size and capillary pressure is depicted in Equation (5), where the *R* is radius, γ is the surface energy of water, and θ is the contact angle. This function approximately determines whether the water can penetrate the pore inlet, and it is difficult to reflect the internal water conditions of the porous GDL. The capillary pressure, *P*_c_, was calculated using Equation (6):

(6) $$P_{c}=\gamma cos\theta {\left(\frac{\varepsilon }{\kappa }\right)}^{0.5}J\left(s\right)$$

(7) $$K=\frac{\varepsilon^{3}\frac{1}{4}{\sqrt{\frac{4}{\pi }dp}}^{2}}{96{\left(1-S\right)}^{2}}$$

where *K* is the permeability determined by Equation (7), and *J*(s) is the Leverett *J*‐function related to the water saturation. Previous studies^[^ ^119^, ^120^ ^]^ showed the relationship between capillary pressure and saturation for the GDL. Conventionally, with an increase in the inlet pore size, the necessary penetration pressure is reduced and less work is required to overcome the unfavorable surface energy.^[^ ^19^, ^121^ ^]^ The cracks on the surface of the MPL have a function similar to that of natural large pores, both of which are beneficial to water removal.^[^ ^122^ ^]^ Further optimization of the surface and inside structure of DGL is artificially performed to enhance water transport, namely, perforation.^[^ ^17^, ^18^, ^123^, ^124^ ^]^ These works concluded the depth, position, and morphology of perforation mentioned in the preceding section. Among them, the perforation depth is difficult to evaluate and is completely determined by the difference in the pressure or capillary pressure gradient. As mentioned earlier, Chen et al. designed various surface formations for MPL and exhibited point‐, line‐, and flower‐like patterns.^[^ ^92^ ^]^ Liquid water is compressed at the gas outlet, providing a larger active region for the reaction. This optimization of the internal and interfacial structures concerns the internal transport of water because the liquid water intruding into the smaller pores is difficult to expel.^[^ ^116^ ^]^ Generally, liquid water transport in the passages of the GDL occurs in two forms: continuous flow^[^ ^125^ ^]^ and discrete droplets^[^ ^126^ ^]^ (**Figure** **9a**), which are usually distinguished by the driving forces of capillary pressure; however discrete droplets are also commanded by inertia, viscosity, gravity, and evaporation forces. Using simulations to investigate water motion, several dimensionless numbers, i.e., Microscale Reynolds numbers (Re), capillary number (Ca), and Bond number (Bo),^[^ ^127^, ^128^ ^]^ were chosen to depict the primary displacement patterns. Re the represents the ratio of inertial force to viscous force, Ca denotes the ratio of viscous force to surface tension, and Bo describes the ratio of the gravitational force to surface tension, which the actual numbers among them are of the order of 10^−5^, 10^−7^, and 10^−5^, respectively. In general, viscous and gravitational effects are sufficiently small and can thus be ignored. However, the Ca of the MPL and MPS varies substantially, causing two flow regimes: capillary fingering and viscosity fingering, which explains why the small pores are invaded with difficulty.^[^ ^127^ ^]^

According to Equation (5), the water penetration and percolation are related to the surface tension. At the MPL/CL interface, surface tension or hydrophobicity prevents water intrusion into the inlet. The MPL/MPS interface is similar to the MPL/CL interface; however, there is little distinction between the existing large capillary difference pressures at this interface. Most perspectives are divergent for the role of hydrophobicity and hydrophilicity. More investigations on the roles of hydrophobicity and hydrophilicity are divergent. Other investigations have reported that a hydrophobic GDL boosts water removal.^[^ ^95^, ^132^ ^]^ Others hold that hydrophilic pores transport water easily and that a hydrophilic solid can lead to water flow.^[^ ^121^, ^133^ ^]^ The wettability of carbon is usually neglected; consequently, the mass ratio of PTFE to carbon particles or fibers should be considered.^[^ ^110^ ^]^ However, the wettability of the GDL is affected by the distribution of hydrophobic agents, such as PTFE. Vacuum drying can form a more uniform PTFE distribution than air drying.^[^ ^20^ ^]^ Chen et al. also found that high contents of PTFE accumulated at the intersection of carbon fibers with the air‐drying treatment.^[^ ^92^ ^]^ Clustered PTFE leads to the collection of water, and further causes non‐uniform current density distribution. Li et al. constructed a 3D regular pore network modeling (PNM) to investigate the influence of spatially variable wettability on water and oxygen transport. Generating four types of hydrophilicity configuration: uniform configuration, symmetric graded configuration, positive and negative graded configuration, the intrusion of hydrophilicity pores divided two areas: low and high hydrophilic regions, where the hydrophilic pore fraction (0.4) was the boundary. The GDL with a positively graded configuration exhibits a higher limiting current density than the others. Subsequently, they showed that oxygen pushed liquid water to advance along the through‐plane direction, resulting in a uniform water distribution, which improved the performance of fuel cells.^[^ ^134^ ^]^ Hou et al. investigated water motion on the superhydrophilic side and top walls of GDL (Figure 9b). This is a novel water management method. The liquid water droplet was absorbed and covered the side and top walls in a pattern of thin‐film shape, which was easily blown out of the flow channel or evaporated.^[^ ^130^ ^]^ Niblett et al. also reported that the water droplets attached to the hydrophilic channel walls easily enhance water droplet detached.^[^ ^131^ ^]^ Receding of interfaces was observed due to the detachment of water from the GDL, which reduced the water saturation and capillary pressure (Figure 9c). A stable liquid water column was provided by an ordered anisotropic structure, similar to the tendril model.

**Figure 10:**
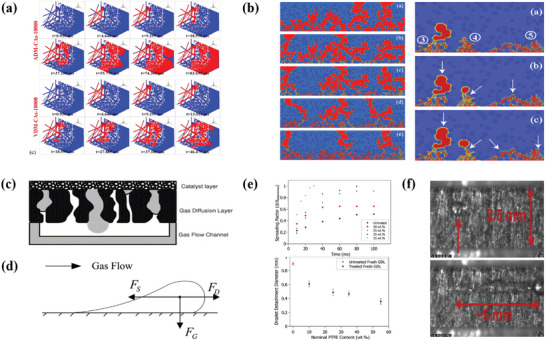
a) Invading process of liquid water inside GDL with MPL as time evolves (air‐drying and vacuum‐drying); and variations of water saturation along GDL thickness direction at different time moments, as well as air‐drying and vacuum‐drying when GDL/MPL simulates at a pressure difference of 10 000 Pa. Reproduced with permission.^[^ ^20^ ^]^ Copyright 2019, Elsevier. b) Steady‐state distribution of liquid water clusters in PTLs with different thicknesses of MPL; and dynamic invasion behavior of the liquid water clusters in a PTL with an MPL intrusion thickness of 50 mm. Reproduced with permission.^[^ ^128^ ^]^ Copyright 2014, Elsevier. c) Water in the outlet of the gas diffusion layer. Reproduced with permission.^[^ ^116^ ^]^ Copyright 2005, Elsevier. d–f) Forces applied on a droplet in general configuration; droplet growth and detachment under fast facial air velocity of 11.1 m s^−1^; droplets slid on untreated GDL without detachment. Reproduced with permission.^[^ ^25^ ^]^ Copyright 2013, Elsevier.

With the introduction of a PTEF or MPL, a porosity gradient from the inlet to the outlet of the water^[^ ^26^, ^124^, ^133^ ^]^ and different distributions of wettability^[^ ^134^, ^135^ ^]^ can be realized. Currently, most of the structural hypotheses that affect water transport in the GDL, are evaluated using different diagnostic tools, this is numerical models, such as the volume of fluid (VOF), pore network modeling (PNM), and the lattice‐Boltzmann method (LBM). Liu et al. believed that the polarization effect substantially affected the droplet motion in complex spatial electric fields. They developed a 2D isothermal VOF method that described the polarization force relatively precisely. The results showed that while the water droplet contacted the fibers of different layers simultaneously, voltage gradients with the droplet were generated, which impacted the growth and shape of water clusters, and further changed their subsequent motion path and rate. Unfortunately, this influence was weaker than that of pore distribution. However, it can reduce the pressure difference between the interior and outside of the droplet to provide a greater driving force.^[^ ^136^ ^]^ Subsequently, a 3D LBM was developed by Deng^[^ ^20^ ^]^ to study the liquid water motion and reconstruction at the MPS/MPL interface. With the MPL insertion, water easily intrudes into the MPS because of the considerable pore size difference, causing a large penetrating capillary pressure difference (**Figure** **10a**). Liquid water droplets search for an optimal passage through which to percolate and form large water clusters. Using the SC‐LBM model, Kim et al. found that the greater the intrusion of the MPL and PTFE (i.e., with a thick intrusion area in the through‐plane) the less water percolated into the MPS (Figure 10b).^[^ ^128^ ^]^ When the water crosses the TP direction the of GDL, it forms a complete water tendril model in the GDL.^[^ ^137^, ^138^ ^]^ In the early 21st century, Nehlsen et al. mentioned that once the percolation path was built, the water on the back hardly spent any work overcoming the surface energy, which was a prototype of the water tendril model.^[^ ^116^ ^]^ While water was transported through the tendril model, there was no further intrusion of liquid water into void pores to form a stable liquid‐phase flow;^[^ ^138^ ^]^ Accordingly, the oxygen transport resistance was constant in this region. If the water droplet breaks through the outlet, there must be large pores to decrease the capillary pressure; otherwise, water will be trapped in the cavities (Figure 10c).

The different PTFE contents and superficial gas velocities also affected the water droplet growth and detachment (Figure 10d). Mortazavi et al. defined a spreading factor, the ratio of the droplet diameter d to the droplet detachment diameter dd, to reveal the relationship between the PTFE content and the water droplet detachment diameter. The higher the PTFE content, the less time was needed to reach the detachment diameter, this is a short steady‐state time of liquid water and gas (Figure 10e,f).^[^ ^25^ ^]^ However, relative to the gas velocity, the detachment diameter was not a strong function of the PTFE content within the GDL. Subsequently, with the droplet growth at the GDL surface, it does not detach naturally, but is the interaction result of the surface adhesion force and drag force applied from the gas flow. Generally, a high gas velocity is conducive to the water detachment, but leads to low reactant utilization and possibly membrane dehydration, too. As previously mentioned before, the wall effect of the flow channel is significant for the water detachment. When the large pores were present under the channel, the liquid water emerged directly into the flow channel. While the pores were under the land, water moved along the interface between GDL and the land of bipolar plate and subsequently, wicked onto the channel side walls.^[^ ^139^ ^]^

However, the water transport behavior of PEMWE has not yet been thoroughly investigated. Qualitative analysis results of the relationship between GDL structure and water transport through experiments and model simulations.^[^ ^5^ ^]^ For example, Zinser et al. established a spatial distribution model for the gas‐liquid transport process to predict the water and gas distributions. The results showed that high porosity, high permeability, and a low contact angle were suitable for maintaining a sufficient water supply.^[^ ^140^ ^]^ Han et al. also found that the increase in water saturation when the porosity increased from 35% to 55% was due to an increase in water permeability, which increased the limited current density and reduced the cell overpotential.^[^ ^141^ ^]^ Thus, there is a water transport difference between PEMWE and PEMFC. Temporarily, the difference in water transport of GDLs between PEMFC and PEMWE can be obtained from the wettability of the fuel cells, which are usually designed to be hydrophobic; instead, electrolytic cells require hydrophilicity to ensure water supply. Of course, proper structural design is necessary for PEMWE and PEMFC, which usually depend on clear water transport mechanisms. However, despite numerous investigations on the water transport mechanisms in the GDL, the practical and specific mechanism of water transport in PEMFC and PEMWE remains obscure. Some of the micropores can be manufactured by compression and adding PTFE in the GDL of PEM fuel cell, which is hardly evaluated from visualization technologies with low solution, accordingly. State‐of‐the‐art computer‐aided simulation and finite element analysis are beneficial and strong tools to overcome this defect at this time. It is also suggested that researchers should pay attention to the transport of water when exploring bubble motion in the GDL of PEMWE.

## 4 Challenges and Recommendations

PEMWE and PEMFC exhibit great potential in the hydrogen energy industry. Recently, the research focus has shifted to the key components, viz. the membrane and catalyst. Although the GDL is an effective masstransfer medium, research on its application in both PEMFC and PEMWE is still in its infancy, and some material, structure, and mass_−_transfer issues remain unresolved. Two electrochemical devices are required to address their own problems because of their various operational environments and work objectives. For PEMWE, a large part of the improvements serve the durability of the GDL; however, the results are not satisfactory due to its highly corrosive environments, in which the proper materials show the best performance and a set of conditions are not definitively determined. Thus, solving material issues is urgent, and further evidence of GDL‐induced corrosion has not been reported to date. There is no further evidence that the GDL causes corrosion. Therefore, the targeted measures could not be implemented. Although Ti is the most conventional GDL material, researchers are still actively exploring new materials for use in PEMWE. Carbon fibers were used on the anode GDL of the PEMWE. Certainly, limited by the high positive potentials and extreme oxidative environments, this type of GDL is deformed and damaged after a short time; accordingly, it is difficult to directly apply the results of fuel cells on PEMWE to the carbon fiber materials is difficult. Some metal GDLs will also be corroded if there are no protective layers; other components such as the catalyst layer and membrane are easily poisoned, and iron cations may contaminate the MEA. However, the conventional method to counter the cationic contamination of the membrane and the catalyst layer, replacing the stainless steel with polymer materials and using ion exchangers to maintain high‐purity water is only employed in the low‐pressure electrolysis, because materials of high‐pressure components are hard to substitute. The cost of materials and production is a challenge that must be considered for large‐scale commercial applications. In the PEMWE the coatings with large content of precious metals are only used on a laboratory scale, while scaling up to the industry, it has to consider the costs of production. And titanium itself is more expensive than other commonly used metal materials, such as stainless steel. Besides, there are many methods to prepare GDL but few of them can expand to the industry scale in that they possess the same defects, for example, vacuum plasma spraying is a low‐cost and scalable technology but one challenge is the use of vacuum. Carbon nanotubes have been taken as the alternative materials for the MPL in the fuel cell, showing excellent electrical conductivity and electrochemical performance, and naturally, the material costs are high. Electrospun and metal foam gas diffusion layer substrates are considered great promising components for the PEMFC industry, allowing for control over the structure and physical properties of GDL. So, the costs of materials and technologies are the key to whether the GDL is suitable for deployment on a large scale.

The GDL is a porous component and the limited available structures make it most underexplored in both PEMFC and PEMWE. First, the architectural design of the GDL is a substantial challenge, whose designs should consider the transfer of both electrons and thermal and water vapor transport behaviors, especially the removal of products for fuel cells and electrolyzers. The typical uniform porosity and pore distribution of MPS cannot be achieved easily, especially after the addition of MPL. Subsequently, gradient designs were considered to improve performance. Therefore, a non‐uniform structure with a pore gradient is conducive. The only information acquired is that the microstructure of the GDL is related to the two‐phase flow. Unfortunately, the lack of accurate data on the related structural parameters of GDL affects the details of the water and gas transport mechanisms awareness. Two difficulties impede the improvement of mass transport loss. First, the micropores in the MPL could not be accurately characterized and the behavior of two‐phase flow could not be observed in the GDL, particularly at the GDL/CL interface. The latest design is the transparent structure of GDL,^[^ ^115^, ^142^ ^]^ which allows the observation of water and gas conditions using only a high‐speed camera. This visualization system can visualize the interface at different spatial and temporal scales. For conventional non‐transparent GDL, visualization technologies limited by their resolution make it difficult to reconstruct their structures. Compared to neutron and optical imaging the XCT has developed the nano‐ to sub‐micron size,^[^ ^143^, ^144^ ^]^ which greatly promoted research on the specific connection between microstructure and the condition of water and revealed many valuable insights. Computational simulation is a popular method and an effective tool for studying the transport properties in the GDL; it can predict and instruct experiments as complementary to experimental measurements. Although significant progress in simulation modeling has been achieved in recent years, the accurate simulation of the liquid water and gas transport progress and state accurately remains a significant challenge. First, the stochastic reconstructed structure cannot reflect a realistic porous structure and the reconstructed microstructure is divided into a large number of grids, resulting in slow computational speed and limited computational simulation capacity. Therefore, researchers may be able to focus on this area. Few investigations have been concluded to solve reconstruction and two‐phase flow models in an integrated simulator. In addition, the choice and simplification of various models are difficult based on the relative research goals. In short, the two PEM electrochemical applications (i.e., PEMFC and PEMWE) have significant potential and are considered to be one of the best methods for reducing anthropogenic carbon emissions. Therefore, when research is continued in the laboratory, commercial applications on a large scale should also pick up the pace, and thus attract increased financial investment.

## 5 Conclusion

PEMFC and PEMWE are the typical applications of PEM for the “hydrogen economy”, which assemble similar components. The GDL is the key component for achieving gas and water transports. According to different chemical reactions and application conditions, their materials, structures, and wettability differ, and they exhibit different mass transfer states. Thus, it is difficult to apply the carbon materials to the anodes of PEMWE because of their high anode potentials. Metal substrates are also prone to corrosion; therefore, a protective layer is usually added to prevent the degradation of the MPS. However, these costs have become a significant problem for electrolyzers. Hence, reducing the cost is a greater effort. To reduce the performance loss, proper structural designs and wettability distribution need to be considered. Acquiring pore sizes and building porosity gradients (i.e., forming multilayer structures) tend to be the direction of attempts. Gas and water intrude different pores: gas is affected by “bubble effects”, the distribution of pore size could not be uniform and the pore sizes cannot be too large (<50 µm) at the CL sides in PEMWE. Hydrophilic GDLs are suitable for gas growth and separation in PEMWE. In this review water transport in the GDL of PEMFC is divided into three processes: inlet intrusion, internal transport, and outlet separation. These processes require different pore structures and hydrophobicity distributions; the inlet position needs to absorb water from the CL/GDL interface, so the pore sizes must be bigger, and the pores need to show hydrophilicity. Within the internal GDL, the smaller pores can produce a large driving force that promotes water transport. At the outlet, the pore size increases again to prevent water preservation in the small pores. However, the transportation of water in WE has not been deeply discussed, so we hope to make some explorations in this regard. Finally, this paper also mentions the current challenges of both devices, respectively, which indicates the key points of research and how to absorb strength from each other by comparing different aspects of the design of next‐generation PEM devices.

## Conflict of Interest

The authors declare no conflict of interest.

## Author Contributions

T.Z. wrote the original draft. L.M. performed the conceptualization, writing review & editing. C.C. performed the investigation. L.D. and performed the writing review & editing. L.X. and C.T. performed the writing review. J.H. performed the project administration. S.Y. performed the supervision.
